# Long Noncoding RNA LOC550643 Acts as an Oncogene in the Growth Regulation of Colorectal Cancer Cells

**DOI:** 10.3390/cells11071065

**Published:** 2022-03-22

**Authors:** Hsuan Franziska Wu, Tzung-Ju Lu, Yi-Hao Lo, Ya-Ting Tu, Yi-Ru Chen, Ming-Cheng Lee, Yu-Lun Chiang, Chung-Yu Yeh, Kuo-Wang Tsai

**Affiliations:** 1 Department of Family Medicine, Zuoying Branch of Kaohsiung Armed Forces General Hospital, Kaohsiung 81342, Taiwan; jimmyrehab@vghtc.gov.tw (H.F.W.); a1090277@g-mail.nsysu.edu.tw (Y.-H.L.); 2 Division of Colon and Rectal Surgery, Department of Surgery, Taipei Tzu Chi Hospital, Buddhist Tzu Chi Medical Foundation, New Taipei City 23142, Taiwan; ndmcnalien@tzuchi.com.tw; 3 Department of Marine Biotechnology and Resources, National Sun Yat-sen University, Kaohsiung 80424, Taiwan; 4 Institute of Medical Science and Technology, National Sun Yat-sen University, Kaohsiung 80424, Taiwan; 5 Department of Research, Taipei Tzu Chi Hospital, Buddhist Tzu Chi Medical Foundation, New Taipei City 23142, Taiwan; tch33241@tzuchi.com.tw (Y.-T.T.); xd802151@tzuchi.com.tw (Y.-R.C.); xd108221@tzuchi.com.tw (M.-C.L.); tch33843@tzchi.com.tw (Y.-L.C.); 6 Department of Medical Education and Research, Kaohsiung Veterans General Hospital, Kaohsiung 81362, Taiwan; cyyeah@vghks.gov.tw

**Keywords:** LncRNA, microRNA, LOC550643, miR-29b, colon cancer

## Abstract

Long noncoding RNAs play a key role in the progression of colorectal cancer (CRC). However, the role and mechanism of LOC550643 in CRC cell growth and metastasis remain largely unknown. In this study, we assessed the clinical impacts of LOC550643 on CRC through the analysis of The Cancer Genome Atlas database, which revealed the significant upregulation of LOC550643 in CRC. Moreover, the high expression of LOC550643 was associated with poor survival in patients with CRC (*p* = 0.001). Multivariate Cox regression analysis indicated that LOC550643 overexpression was an independent prognostic factor for shorter overall survival in patients with CRC (adjusted hazard ratio, 1.90; 95% confidence interval, 1.21–3.00; *p* = 0.006). A biological function analysis revealed that LOC550643 knockdown reduced colon cancer cell growth by hindering cell cycle progression. In addition, LOC550643 knockdown significantly induced cell apoptosis through the inhibition of signaling activity in phosphoinositide 3-kinases. Moreover, LOC550643 knockdown contributed to the inhibition of migration and invasion ability in colon cancer cells. Furthermore, miR-29b-2-5p interacted with the LOC550643 sequence. Ectopic miR-29b-2-5p significantly suppressed colon cancer cell growth and motility and induced cell apoptosis. Our findings suggest that, LOC550643–miR-29b-2-5p axis was determined to participate in the growth and metastasis of colon cancer cells; this could serve as a useful molecular biomarker for cancer diagnosis and as a potential therapeutic target for CRC.

## 1. Introduction

The GLOBOCAN 2018 indicates that colorectal cancer (CRC) is the second most lethal cancer in the world, accounting for approximately 9.2% of cancer-related deaths, or 881,000 deaths. Estimates for new CRC cases in 2018 exceeded 1.8 million, making CRC the third most common cancer that year [1]. Both hereditary and environmental factors, including increased body mass index, alcohol consumption, tobacco smoking, low vitamin D intake, and certain cooking methods (frying, boiling, and charcoal broiling), influence CRC risk [2]. In clinical settings, the selection of therapeutic modalities depends on the size, location, and pathological stage of the tumor. New treatment options, such as triplet chemotherapy, biological therapy, salvage therapy, and immunotherapy, have improved overall survival rates in patients with advanced CRC. However, survival rates remain higher when no metastasis is involved [3]. Therefore, to reduce the morbidity and mortality of CRC, early detection and novel therapeutic targets are urgently needed.

Most of the human genome does not encode protein sequences; protein-coding genes account for <2% of the entire genome [4]. Numerous studies have demonstrated that noncoding RNAs (ncRNAs) play a pivotal role in several biological reactions, including cancer progression [5,6,7,8,9,10]. According to transcript length, ncRNAs can be classified as short ncRNAs (microRNA) or as long ncRNAs (lncRNA), which are RNA transcripts that are more than 200 nucleotides long. Studies have reported that lncRs play a role in the regulation of various cellular processes [9,11]. Mounting evidence indicates that numerous lncRs, including BANCR, CCAT1, HOTAIR, H19, MALAT1, PCAT-1, PVT-1, LOC441461, and 91H, participate in CRC. These dysfunctional lncRNAs have been reported to have tumor-suppressive or oncogenic roles in numerous cellular processes in CRC, including cell growth, the cell cycle, and cell migration [6,8,12,13,14,15,16,17,18,19,20,21,22,23]. Our previous studies have revealed that LOC550643 expression is significantly elevated in breast cancer cells and is involved in their growth and motility [24]. However, the role of LOC550643 remains largely unknown, and mechanisms concerning dysfunctional lncRNAs in CRC have not yet to be elucidated. Herein, we investigated the clinical effects of LOC550643 and its biological function in colon cancer.

## 2. Materials and Methods

### 2.1. Cell Lines and Clinical Samples

Colon cancer cell lines, namely colo205, DLD-1, HCT116, LS174T, and LoVo, were obtained from the American Type Culture Collection. Cells were cultured in high-glucose Dulbecco’s modified Eagle’s medium (Invitrogen, Grand Island, NY, USA) and supplemented with 10% fetal bovine serum (Hyclone Laboratories, Inc., South Logan, UT, USA) and 1% penicillin/streptomycin (Gibco, Thermo Fisher Scientific Inc., Waltham, MA, USA). Next, cells were cultured at 37 °C and supplied with 5% CO_2_. Furthermore, we obtained five samples of histologically normal tissue adjacent to tumors in patients with CRC, which were collected by the biobank of Taipei Tzu Chi Hospital (New Taipei City, Taiwan). Informed consent was obtained from all patients by the biobank. The study protocol was approved by the Ethics Committee of Taipei Tzu Chi Hospital (09-X-008).

### 2.2. RNA Extraction

Total RNA was extracted from cells and tissues by using TRIzol reagent (Invitrogen, Grand Island, NY, USA). The tissue samples were first subjected to homogenization with 1 mL of TRIzol reagent. Subsequently, the protein was removed using 0.2 mL of chloroform. Finally, total RNA was precipitated using 0.5 mL of isopropanol. Total RNA concentration was measured on a Nanodrop 1000 spectrophotometer (Nanodrop Technologies Inc., Wilmington, DE, USA).

### 2.3. Expression Data from Public Databases

The RNA expression profiles of 480 colon cancer tissue samples and histologically normal tissues adjacent to the tumors were obtained from The Cancer Genome Atlas (TCGA) data portal (https://tcga-data.nci.nih.gov/tcga/dataAccessMatrix.htm, accessed on 29 January 2018). Clinical data on the patients with CRC were also obtained from TCGA. We considered an additional cohort, examining LOC550643 expression in 3775 colon cancer tissue samples and 397 histologically normal tissues that were obtained from the Gene Expression database of Normal and Tumor tissues (GENT; http://gent2.appex.kr/gent2/ accessed on 8 February 2022) [25].

### 2.4. Gene Ontology and Pathway Enrichment Analysis

The LOC550643 associated genes were analyzed using g:Profiler online tool (http://biit.cs.ut.ee/gprofiler/gost accessed on 8 February 2022) [26], which may be used for the high-throughput functional analysis of genes. The g: Profiler database includes GO and KEGG pathway analyses, which are useful tools for illustrating putative biological information.

### 2.5. Reverse Transcription and Real-Time Polymerase Chain Reaction

Total RNA (2 μg) was obtained from the cell and tissue samples. Next, cDNA was subjected to reverse transcription by using the SuperScript IV Reverse Transcriptase kit (Invitrogen; Thermo Fisher Scientific Inc., Waltham, MA, USA) and random primers (Invitrogen; Thermo Fisher Scientific Inc.). Subsequently, cDNA was subjected to individual gene expression analysis through real-time polymerase chain reaction (PCR). Gene-specific primers were employed for individual gene amplification, and a Fast SYBR Green Master Mix (Applied Biosystems; Thermo Fisher Scientific Inc.) was used for PCR product detection. The primers sequences used are listed as follows:

LOC550643-F: 5′-GGAGATCCACTCTCCCACCTGGAAA-3′

LOC550643-R: 5′-GACTCCATTTCCCTGGTGCATTCA-3′

GAPDH-F: 5′-TGCACCACCAACTGCTTAGC-3′

GAPDH-R: 5′-GGCATGGACTGTGGTCATGAG-3′

miR-29b-2-5p-RT: 

5′-CTCAACTGGTGTCGTGGAGTCGGCAATTCAGTTGAGCTAAGCCA-3′

Universal primer: 5′-CTGGTGTCGTGGAGTCGGCAATTC-3′

miR-29b-2-5p-GSF: 5′-CGGCGGCTGGTTTCACATGGTG-3′

U6-F: 5′-CTCGCTTCGGCAGCACA-3′

U6-R: 5′-AACGCTTCACGAATTTGCGT-3′

### 2.6. Cell Proliferation Assay

Lipofectamine RNAiMAX (Invitrogen; Thermo Fisher Scientific Inc.) was used to transfect siLOC550643 (siRNA#301 and siRNA#543), miR-29b-2-5p mimics, or a scrambled control into colon cancer cells. Following transfection with siRNA or the mimics, 1000 colon cancer cells were seeded into 96-well plates. At different time points (0, 1, 2, 3, and 4 days), cell proliferation ability was examined using the CellTiter-Glo One Solution Assay kit (Promega Corporation, Madison, WI, USA). Detailed information on the procedure can be found in our previous study [24].

### 2.7. Colony Formation Assay

Colon cancer cells transfected with siLOC550643, miR-29b-2-5p mimics, or a scrambled control were seeded in six-well plates (2000 cells/well) and incubated in culture medium containing 10% fetal bovine serum at 37 °C for 2 weeks. Subsequently, the cell colonies were fixed with 4% formaldehyde and stained with crystal violet solution. Colony formation was observed through microscopy. To solubilize the crystal violet color of the colonies, 1 mL of 10% acetic acid was added. Colony formation ability was evaluated using a spectrophotometer at a wavelength of 595 nm. Detailed information on the procedure can be found in our previous study [24].

### 2.8. Cell Migration and Invasion Assay

For migration abilities assays, colon cancer cells were seeded into a the upper chamber of transwell chambers containing 8-um pores (Corning Costar, Lowell, MA, USA). For invasion assay, cells were added to the upper chamber which was coated with 50 ug/mL of type I collagen or 80 µg of Matrigel. After incubation for 24 h at 37 °C, the cells at the lower side were stained using Giemsa. The level of migration or invasion was determined using a microscope at 200× magnification. All experiments were repeated three times.

### 2.9. Cell Cycle Progression Analysis

For cell cycle progression analysis, colon cancer cells were mixed with 70% ethanol. After staining with 4′,6-diamidino-2-phenylindole (ChemoMetec, Gydevang, Lillerød, Denmark), cells were examined through imaging flow cytometry, and the population of cells in each phrase was evaluated using NucleoView NC-3000 software (ChemoMetec). Detailed information on the procedure can be found in our previous study [24].

### 2.10. Cell Apoptosis Analysis

After siLOC550643, miR-29b-2-5p mimics, or a scrambled control were transfected into colon cancer cells for 48 h, the cells was stained with CF488A Annexin V and PI Apoptosis kit (Biotium, Fremont, CA, USA). Finally, apoptotic colon cancer cells were detected through image flow cytometry analysis, and the population of apoptosis cell was quantified using NucleoCounter NC-3000 (ChemoMetec, Gydevang, Lillerød, Denmark) and NucleoView NC-3000. Detailed information on the procedure can be found in our previous study [24].

### 2.11. Western Blotting

After 24 or 48 h of transfection with siLOC550643, miR-29b-2-5p mimics, or a scrambled control, protein lysate was obtained using lysis buffer. Next, protein content in the cell lysate was determined and analyzed according to the manufacturer’s instructions. Detailed information on the primary antibodies employed is provided in Supplementary Table S1. In all experiments, actin served as the loading control.

### 2.12. miR-29b-2-5p Directly Binding at LOC550643 and Luciferase Activity Assay

Binding sites of miR-29b-2-5p on LOC550643 sequence were predicted using the TargetScan tool. The wild type sequences and seed region mutant of LOC550643 were cloned into the pMIR-REPROT^TM^ vector (Invitrogen, Carlsbad, CA, USA). Subsequently, cells were cotransfected with candidate-containing pMIR-REPROT^TM^ vectors and miR-29b-2-5p, or control oligonucleotides by using the Lipofectamine RNAiMAX reagent. Luciferase activity of cell lysates was measured using the Dual-Glo Luciferase Reporter Assay System (Promega, Madison, WI, USA).

### 2.13. RNA Immunoprecipitation Chip Assay

RNA immunoprecipitation chip (RIP) assay was used to examine the interaction of LOC550643 and miR-29b-2-5p using an RNA-binding protein immunoprecipitation kit (Millipore, Billerica, MA, USA). HCT116 cells were transfected with miR-29b-2-5p mimics, or N.C. control for 48 h. The Dynabeads M-280 Streptavidin (Invitrogen, Waltham, MA, USA) conjugated with an anti-Ago2 antibody or anti-IgG were used to perform Ago2 complex precipitation. The Ago2-bounding RNA were extracted using the TRIzol reagent, and the expression levels of LOC550643 or miR-29b-2-5p were examined by real-time quantitative PCR.

### 2.14. Actin Immunofluorescence Staining

Colon cancer cells with LOC550643 knockdown were seeded on coverslips. Cells were fixed and labeled with rhodamine phalloidin, Alexa Fluor 488 DNaseI (1:500; molecular probes), and DAPI. Images were captured using a Zeiss LSM510 Meta confocal microscope. Detailed information on the procedure can be found in our previous study [6].

### 2.15. Statistical Analysis

LOC550643 expression was analyzed using student *t* tests. For the overall survival analysis, the patients were separated into high and low LOC550643 expression groups through a receiver operating characteristic curve to define the optimal cutoff value. The cumulative survival curves of the patients with colon cancer were evaluated through the Kaplan–Meier method. We performed the log-rank test to assess overall survival in patients with colon cancer. All biological functional assays were performed in triplicate. Student *t* tests were conducted to analyze these data, and between-group differences were considered significant at *p* < 0.05.

## 3. Results

### 3.1. Significant Overexpression of LOC550643 in CRC Cells

**Figure 1:**
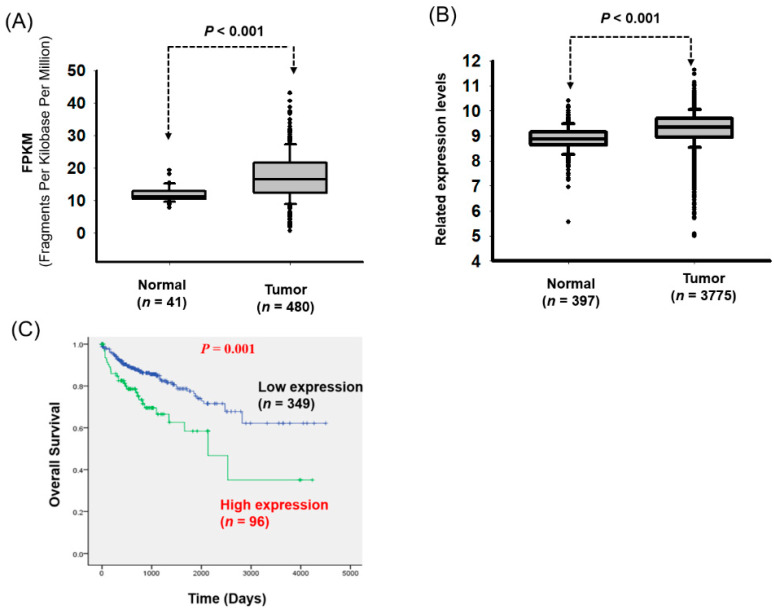
High LOC550643 expression was correlated with poor prognosis in patients with CRC. LOC550643 expression level were examined through the analysis of (**A**) The Cancer Genome Atlas (TCGA) database and (**B**) the Gene Expression database of Normal and Tumor tissues (GENT). (**C**) Kaplan–Meier analysis revealed the association of high LOC550643 expression and poor survival in patients with CRC.

The clinical impacts and biological function of LOC550643 in colon cancer remain unclear. Therefore, we explored the impacts of LOC550643 expression on CRC cells by using a public database. From TCGA, we downloaded the RNA sequencing profiles and clinical information of patients with colon cancer. The data comprised 480 CRC tissue samples and 41 corresponding histologically normal tissues. LOC550643 was significantly overexpressed in the colon cancer cells (*p* value < 0.001, Figure 1A). Using independent cohort analysis revealed that LOC550643 expression was significantly increased in the CRC tissues from GENT database (*p* value < 0.001, Figure 1B). We further analyzed LOC550643 expression in other human cancer types, which revealed the frequent overexpression of LOC550643 in human cancers, including cancers of the bladder, breast, head and neck, liver, kidney and lung cancer (Supplementary Figure S1A,B). These results suggest that LOC550643 may play a crucial oncogenic role in the progression of these human.

We further assessed the clinical impacts of LOC550643 expression, which revealed that the association of high LOC550643 expression with advanced pathological stage (*p* = 0.029) and advanced metastasis status (*p* = 0.004; Table 1). Using the receiver operating characteristic curve, we defined the optimal cutoff value for LOC550643 levels. Based on this cutoff, the patients were divided into high and low LOC550643 expression groups. Kaplan–Meier analysis indicated that high LOC550643 expression was strongly associated with poor overall survival in patients with CRC (Figure 1C). Multivariate Cox regression analysis revealed that high LOC550643 expression is an independent risk factor for worse overall survival (adjusted hazard ratio, 1.90; 95% confidence interval, 1.21–3.00; *p* = 0.006) in patients with CRC (Table 2). In order to explore the putative biological function of LOC550643 in CRC, we attempted to perform the pathway enrichment analysis by g: Profiler approach [26,27]. First, we downloaded the RNA expression profiles of 41 N-T pairs of patients with CRC from TCGA database. By calculating the Pearson correlation between LOC550643 and protein-coding genes in CRC, the negatively and positively co-expressed gene candidates with LOC550643 were identified. A total of 200 protein-coding genes (top of 100 genes with positive correlation and top of 100 with negative correlation with LOC550643 expression) were selected and subjected for pathway enrichment analysis by using g: Profiler. As illustrated in Table 3 and Supplementary Figure S2, these LOC550643 co-expressed genes were significantly enriched in targeting the biological function of protein binding, microfilament motor, cytoskeletal motor, nucleotide-binding, and nucleoside phosphate binding. These results indicated that LOC550643-coexpressed genes are significantly enriched in actin cytoskeleton remodeling regulation, which is a key driver of phenotypic plasticity, cell growth and metastasis in human cancers [28,29].

### 3.2. OC550643 Knockdown Suppressed CRC Cell Growth and Motility

**Figure 2:**
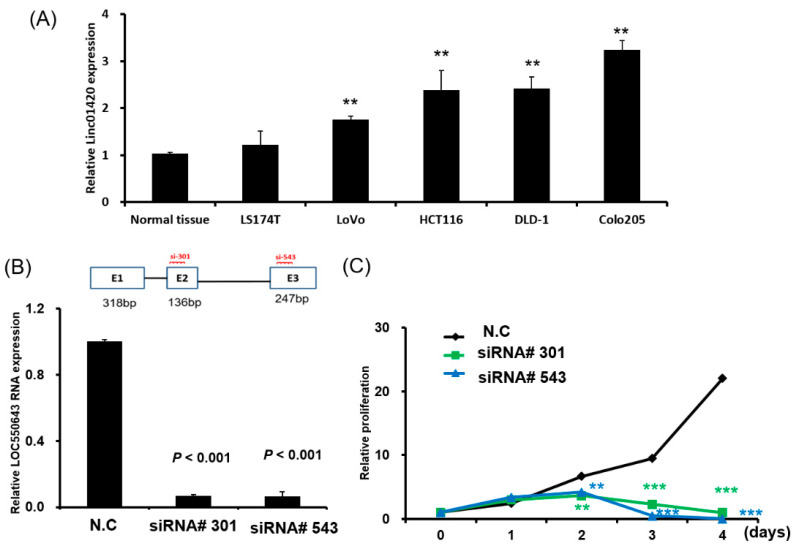
Biological role of LOC550643 in colon cancer cells. (**A**) LOC550643 expression was assessed in five colon cancer cell lines and corresponding histologically normal tissues through real-time polymerase chain reaction (PCR). The normal tissue samples were obtained from five patients with CRC. (**B**) LOC550643 expression levels were examined in HCT116 cells transfected with two siRNAs (siRNA#301 and siRNA#543). (**C**) The cell proliferation assay was conducted on HCT116 cells following their transfection with siLOC550643-#301, siLOC550643-#543, or a scrambled control (** *p* < 0.01, *** *p* < 0.001).

According to the database of the University of California, Santa Cruz, LOC550comprises three exons. In our previous study, we determined the real length of LOC550643 through the 5′ and 3′ rapid amplification of cDNA ends [24] The three isoforms of LOC550643, namely V1, V2, and V3, vary in length (718, 581, and 476 bp, respectively). The role of LOC550643 in the proliferation and metastasis of colon cancer remains unknown. Our examination of LOC550643 expression in five human colon cancer cell lines revealed that LOC550643 was highly expressed in LoVo, HCT116, DLD-1, and Colon 205 cells compared with in the histologically normal tissues (Figure 2A). We further examined HCT116 cells to assess the biological function of LOC550643 by using the loss-of-function approach. Two siRNAs (siRNA#301 and siRNA#543) were used to target exons 2 and 3 of LOC550643, respectively (Figure 2B, upper panel). After the transfection of the individual siRNAs into HCT116 cells over 48 h, LOC550643 expression was significantly reduced in these cells compared with in the control cells (Figure 2B, lower panel). LOC550643 knockdown with siLOC550643-#301 and siLOC550643-#543 significantly reduced HCT116 cell proliferation (Figure 2C). Moreover, this phenomenon was consistently observed in another colon cancer cell line, demonstrating that LOC550643 knockdown significantly suppressed DLD-1 cell growth (Supplementary Figure S3).

### 3.3. LOC550643 Knockdown Hindered Cell Cycle Progression and Induced Apoptosis

**Figure 3:**
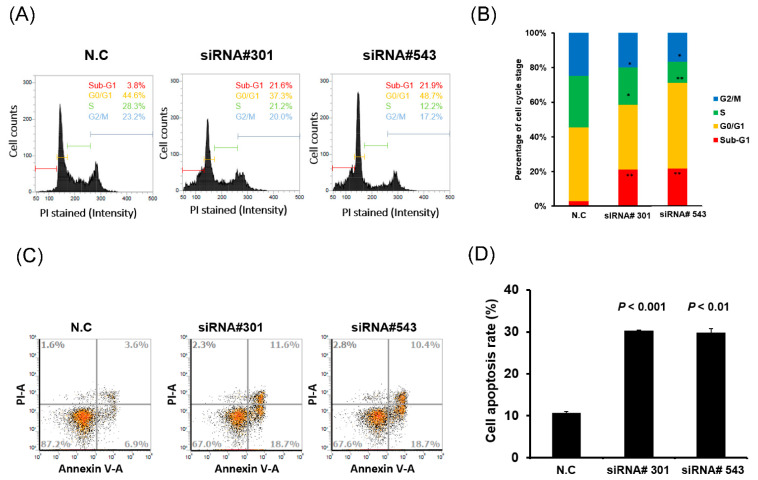
LOC550643 knockdown hindered cell cycle progression and induced apoptosis in colon cancer cells. LOC550643 was knocked down in HCT116 cells through transfection with siRNA-#301, siRNA-#543, or N.C (scrambled control) control. (**A**) Cells in each phase of the cell cycle were analyzed through flow cytometry. (**B**) Cell populations in each phase were quantified using NucleoView NC-3000 software. (**C**) After LOC550643 knockdown for 48 h, cell apoptosis was detected through the annexin V assay. (**D**) The number of apoptotic cells in HCT116 cells with LOC550643 knockdown and in control cells was quantified (N.C group). Experiments were performed in triplicate, and data were analyzed using the student *t* test. Differences were considered significant at *p* < 0.05 (* *p* < 0.05, ** *p* < 0.01).

**Figure 4:**
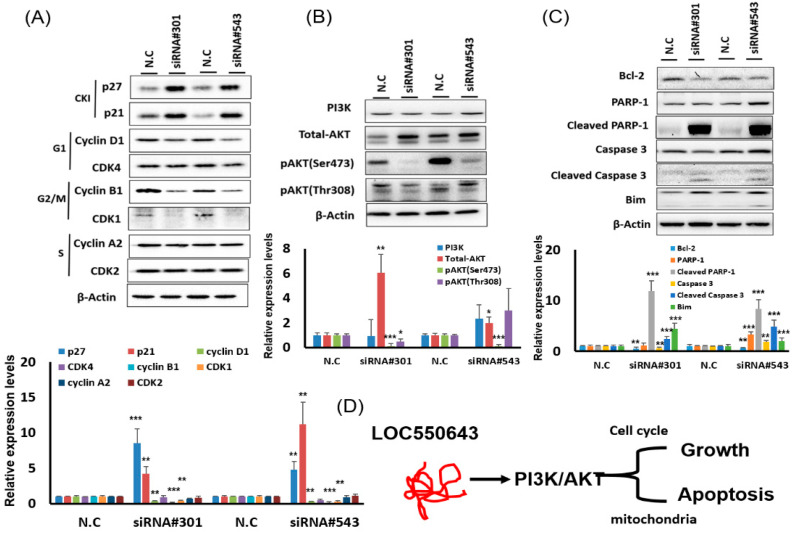
LOC550643 knockdown suppressed colon cancer cell growth through the modulation of phosphoinositide 3-kinase (PI3K) signaling activation. (**A**) Western blotting was conducted to assess the expression levels of cell cycle–related proteins in HCT116 cells with LOC550643 knockdown. Relative expression was further quantification (below panel). (**B**) PI3K signaling-related proteins were examined in HCT116 cells with LOC550643 knockdown. Relative expression was further quantification (below panel). (**C**) Apoptosis-associated protein was assayed in HCT116 cells with LOC550643 knockdown. Relative expression was further quantification (below panel). (**D**) Putative mechanisms of LOC550643 knockdown–induced cell growth inhibition and apoptosis induction were determined (* *p* < 0.05, ** *p* < 0.01, *** *p* < 0.001).

To explore the mechanism by which LOC550643 knockdown contributed to the inhibition of colon cancer cell growth, we performed cell cycle and apoptosis assays. LOC550643 knockdown increased the populations of HCT116 cells in the sub-G1 phase and reduced the populations of cells in the S and G2/M phases (Figure 3A,B). These results suggest that LOC550643 knockdown impaired cell cycle progression and triggered apoptosis in HCT116 cells. Therefore, we examined cell apoptosis in HCT116 cells with LOC550643 knockdown by using the annexin V assay. The number of apoptotic cells increased significantly following 48 h of LOC550643 knockdown (Figure 3C,D). To examine the mechanism by which LOC550643 knockdown was involved in the hindering of cell cycle progression and the induction of apoptosis, we examined the expression levels of cell cycle–related genes and phosphoinositide 3-kinase (PI3K)/AKT signaling–associated protein through Western blotting. The expression levels of cyclin D1, cyclin B1, and CDK1 decreased, whereas those of p27 and p21 increased (Figure 4A). Furthermore, LOC550643 knockdown silenced PI3K activation through the inhibition of AKT phosphorylation (Figure 4B). In addition, the expression levels of Bcl-2 decreased, whereas those of cleaved PARP, cleaved Caspase-3 and Bim increased after LOC550643 knockdown (Figure 4C). In summary, our results indicate that LOC550643 knockdown modulated PI3K signaling activation to suppress cell growth and induce apoptosis (Figure 4D).

### 3.4. LOC550643 Knockdown Suppressed Colon Cancer Cell Motility

**Figure 5:**
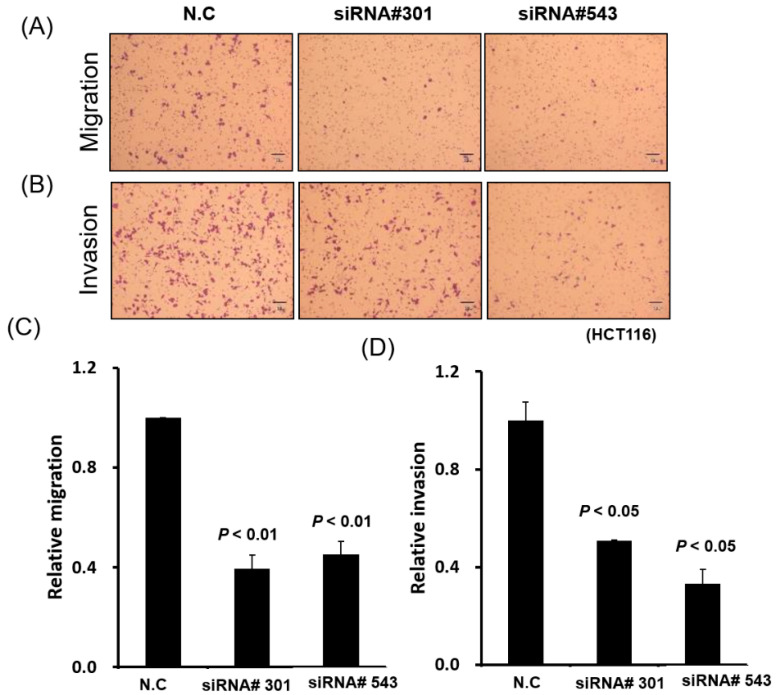
LOC550643 knockdown suppressed colon cancer cell migration and invasion abilities. (**A**,**B**) LOC550643 was knocked down through the transfection of siRNA into HCT116 cells, and their migration and invasion abilities were assessed through the transwell migration assay. (**C**,**D**) Relative migration and invasion abilities were further quantified using Ascent software.

**Figure 6:**
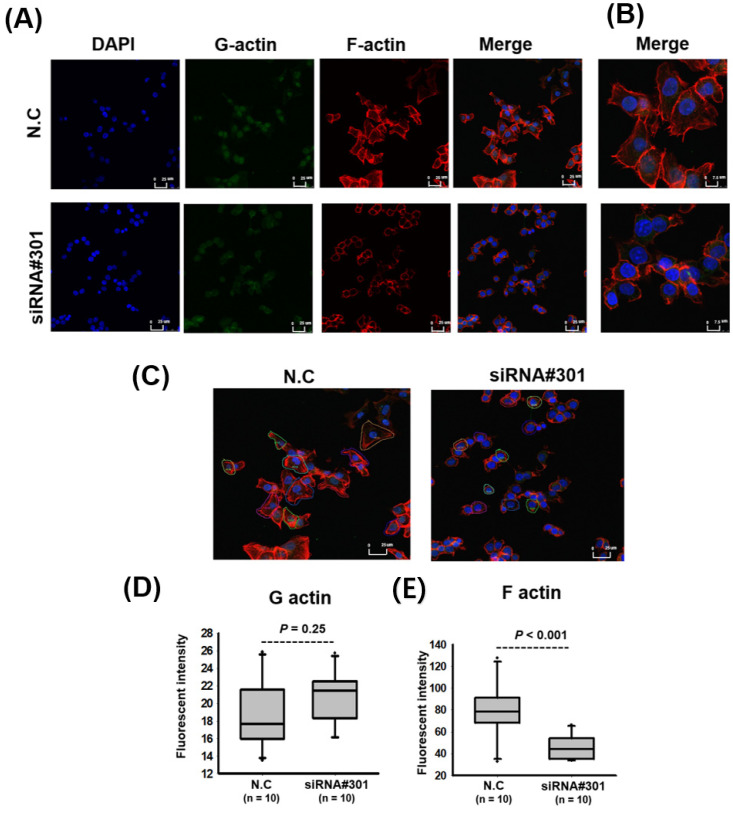
LOC550643 knockdown modulated G-actin and F-actin polymerization to inhibit colon cancer cell motility. (**A**) After LOC550643 knockdown, F-actin and G-actin were stained with rhodamine phalloidin and Alexa Fluor 488, respectively. The fluorescence intensity of HCT116 cells was determined through confocal microscopy (magnification 40×). (**B**) Images of F-actin (red), G-actin (green), and nuclei (blue) were merged, revealing that HCT116 cells with LOC550643 knockdown exhibited fewer filopodium fibers than did normal HCT116 cells (magnification 100×). (**C**–**E**) Fluorescence intensities of G-actin and F-actin were calculated through confocal microscopy (*n* = 10).

As presented in Table 1, high LOC550643 expression was associated with advanced metastasis (Table 1). Therefore, we postulated that LOC550643 knockdown might reduce the motility of colon cancer cells. A transwell migration assay revealed that LOC550643 knockdown significantly inhibited the migration and invasion abilities of HCT116 and DLD-1 cells (Figure 5 and Supplementary Figure S4). In addition, pathway enrichment analysis indicated that LOC550643-coexpressed genes are significantly enriched in actin cytoskeleton remodeling regulation, which is a key driver of cancer cell metastasis. Using immunostaining to examine the levels of G-actin and F-actin indicated that LOC550643 knockdown reduced the generation of cell membrane filopodia protrusions in the colon cancer cells (Figure 6A,B). Further quantification of G-actin and F-actin in colon cancer cells with LOC550643 knockdown was performed through confocal microscopy. The amount of F-actin decreased significantly (Figure 6C–E).

### 3.5. miR-29b-2-5p Inhibited LOC550643 Expression by Directly Binding at Its Sequence

**Figure 7:**
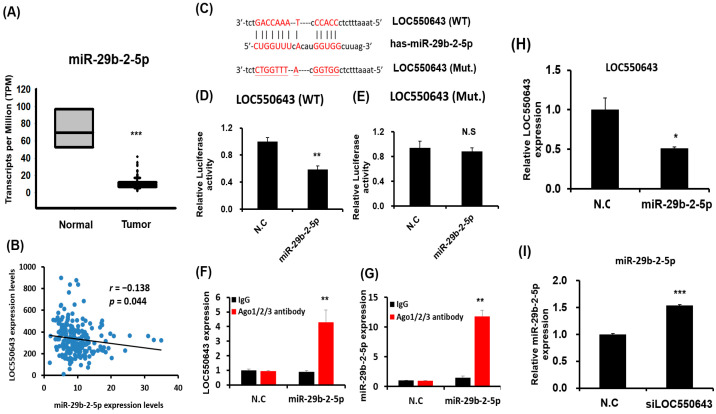
LOC550643 sponged miR-29b-2-5p expression through direct interaction. (**A**) MiR-29b-2-5p expression was examined in CRC cells through the analysis of the TCGA database. (**B**) The relationship between LOC550643 and miR-29b-2-5p in CRC cells was assessed through Pearson correlation analysis. (**C**) Reporter construct sequences with wild-type LOC550643 or a binding site mutant is presented. (**D**,**E**) After miR-29b-2-5p cotransfection with pMIR-REPROT-LOC550643 or pMIR-REPROT-LOC550643_(mutant)_ into colon cancer cells, relative luciferase activity was assessed. Firefly luciferase activity served as a normalization control. (**F**,**G**) HCT116 cells were used to perform Ago2 protein precipitation, and the expression levels of miR-29b-2-5p and LOC550643 were examined using real-time PCR. (**H**) Endogenous LOC550643 expression was examined in HCT116 cells transfected with miR-29b-2-5p mimics. (**I**) Endogenous miR-29b-2-5p expression was assessed in HCT116 cells with LOC550643 knockdown (* *p* < 0.05, ** *p* < 0.01, *** *p* < 0.001).

High LOC550643 expression in CRC tissues was strongly associated with poor patient prognosis. However, the mechanisms underlying abnormal LOC550643 expression and LOC550643 knockdown–induced inhibition of the growth and invasion abilities of CRC cells remain unclear. Assessing the expression of LOC550643 revealed that LOC550643 was mainly accumulated in the cytoplasm of the CRC cells (Supplementary Figure S5). Therefore, we postulated that LOC550643 modulated cell growth and motility by sponging miRNAs expression. We further determined which miRNAs could bind to the LOC550643 sequence by using microRNA prediction tool. We selected miR-29b-2-5p because it was expected to bind to the LOC550643 sequence. Examination of miR-29b-2-5p expression in CRC cells through an analysis of the TCGA database demonstrated that miR-29b-2-5p was significantly reduced in cancer tissue samples compared with in the histologically normal tissue samples (Figure 7A). Furthermore, the Pearson correlation analysis revealed a negative correlation between LOC550643 and miR-29b-2-5p expression in CRC (Figure 7B; *r* = −0.138, *p* = 0.044). The luciferase reporter assay indicated that miR-29b-2-5p suppressed the activity of luciferase activity by directly targeting the LOC550643 sequence (Figure 7C,D). When the binding sites of miR-29b-2-5p were mutated, luciferase activity was restored (Figure 7E). We further demonstrated that LOC550643 interacted with miR-29b-2-3p in vivo through an Ago2 protein precipitation assay (Figure 7F–G). Our results indicated that miR-29b-2-5p overexpression could accumulate LOC550643 in Ago2 complex in HCT116 cells. Furthermore, miR-29b-2-5p overexpression significantly reduced the expression levels of endogenous LOC550643 in the HCT116 cells (Figure 7H). By contrast, LOC550643 knockdown significantly increased the expression of endogenous miR-29b-2-5p expression in the HCT116 cells (Figure 7I). In summary, our present data suggested that LOC550643 might sponge or decoy function on miR-29b-2-5p by directly targeting the LOC550643 sequence.

### 3.6. MiR-29b-2-5p Expression Suppressed Colon Cancer Cell Growth and Induced Apoptosis

**Figure 8:**
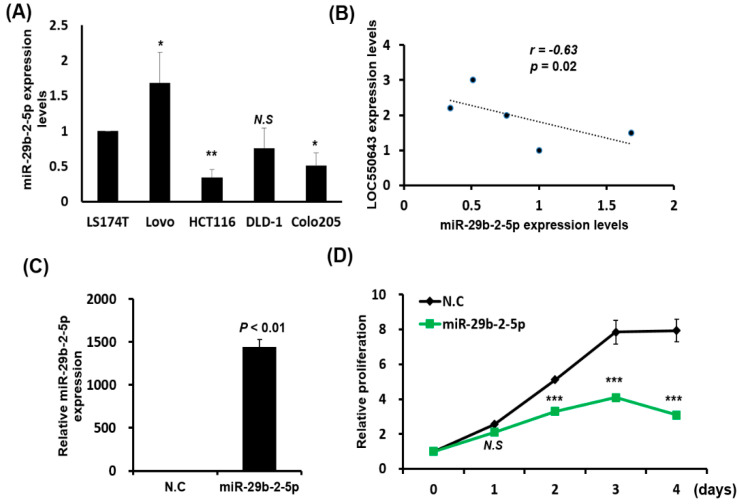

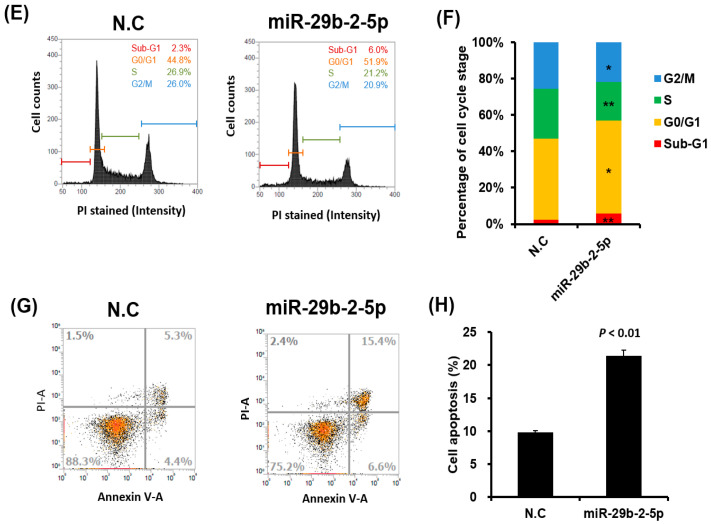
MiR-29b-2-5p overexpression hindered cell cycle progression and induced apoptosis in colon cancer cells. (**A**) The expression levels of miR-29b-2-5p were assessed in human colon cancer cells. (**B**) The correlation between miR-29b-2-5p and LOC550643 were analyzed through Pearson correlation analysis. (**C**) The expression levels of miR-29b-2-5p were examined in HCT116 cells transfected with miR-29b-2-5p mimics over 48 h. (**D**) Cell proliferation was assessed in HCT116 cells transfected with miR-29b-2-5p mimics. (**E**,**F**) The cells in each phase of the cell cycle were analyzed through flow cytometry, and the population of cells in each phase was quantified using NucleoView NC-3000 software. (**G**) Cell apoptosis was detected through the annexin V assay, and (**H**) the population of apoptotic cells in HCT116 cells with miR-29b-2-5p mimic transection and in control cells were quantified (* *p* < 0.05, ** *p* < 0.01, *** *p* < 0.001).

We further assessed the expression levels of miR-29b-2-5p in human colon cancer cells (LS174T, Lovo, HCT116, DLD-1 and Colo205). As shown in Figure 8A, the expression levels of miR-29b-2-5p were high in Lovo cell and low in HCT116 and Colo205 cell compared to LS174T cells. Furthermore, the expression levels of miR-29b-2-5p were negative correlation with those of LOC550654 in human colon cancer cells (Figure 8B; *r* = −0.63, *p* = 0.02). This result reconfirmed our finding that miR-29b-2-5p negatively regulated the expression of LOC550643 in colon cancer. Next, we examined the role of miR-29b-2-5p in colon cancer cells transfected with miR-29b-2-5p mimics through various biological function analyses, namely cell cycle, proliferation, apoptosis, migration, and invasion assays. The miR-29b-2-5p expression significantly suppressed HCT116 cell proliferation (Figure 8C,D). In HCT116 cells transfected with miR-29b-2-5p mimics, the population of cells in the sub-G1 and G0/G1 phases was significantly increased, whereas that of cells in the S and G2/M phases was significantly reduced (Figure 8E,F). In addition, apoptotic cells increased significantly after transfection with miR-29b-2-5p (Figure 8G,H). However, the migration and invasion ability were not significantly change in HCT116 with miR-29b-2-5p (Supplementary Figure S6). We further examined the cell cycle-related genes, PI3K signaling and apoptosis-related genes in HCT116 cell with miR-29b-2-5p expression by western blot. As shown in Supplementary Figure S7, the expression levels of cyclin D1, cyclin B1, CDK2 and CDK4 significantly decreased in HCT116 with miR-29b-2-5p mimic transfection. Furthermore, pro-apoptotic proteins, cleaved PARP, cleaved Caspase-3 and Bim expression levels increased after miR-29b-2-5p overexpression (Supplementary Figure S7B), whereas PI3K/AKT signaling activation did not alter in HCT116 with miR-29b-2-5p mimics transfection (Supplementary Figure S7A). Taken together, our results reveal that miR-29b-2-5p expression mimics the effects of LOC550643 knockdown in colon cancer cells with regard to hindering cell cycle progression and inducing apoptosis, which result in the inhibition of cell growth.

**Figure 9:**
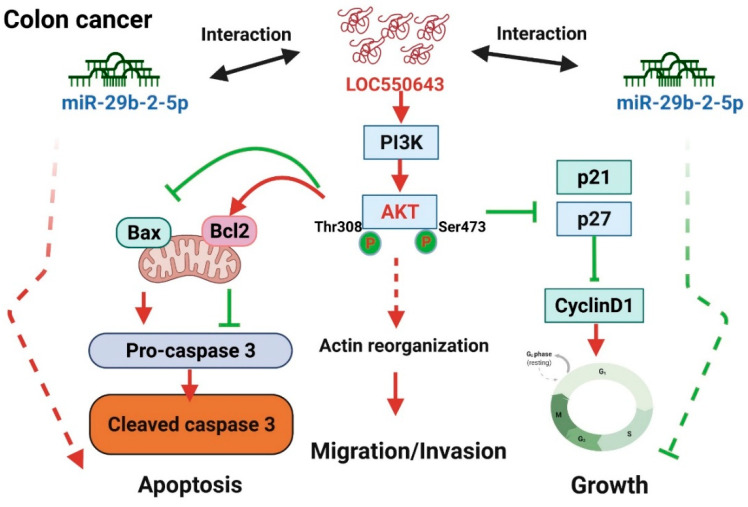
The proposed model for the mechanistic LOC550643 regulation of colon cancer cell growth, apoptosis and metastasis through modulation of PI3K signaling activation and the LOC550643-miR-29b-2-5p axis.

In summary, our current results revealed a novel mechanism that LOC550643 plays an oncogenic role in colon cancer growth and apoptosis through regulating the PI3K signaling pathway and decoying miR-29b-2-5p function (Figure 9). These findings provided valuable insight into the novel oncogenic role of LOC550643, which can serve as a useful prognostic biomarker and therapeutic target in patients with colon cancer.

## 4. Discussion

CRC is a common gastrointestinal cancer. Its diagnosis is reliant on the fecal occult blood test, which is based on detecting gastrointestinal bleeding–induced pseudoperoxidase activity in heme in stool samples. However, the test is unsuitable for prognostic or therapeutic evaluation [30]. Extended *RAS* testing is conducted before the prescription of anti-EGFR therapies in metastatic CRC, but *RAS* mutation is observed only in 30–50% of patients [31]. The literature indicates that lncRNAs play a role in cancer development, regulating the expression of protein-coding genes, participating in epigenetic modification, and modulating alternative splicing processes [32]. Studies have indicated that LOC550643 plays crucial roles in malignancy. LOC550643 is highly expressed in nasopharyngeal carcinoma, thyroid cancer, pancreatic cancer and breast cancer [24,33,34,35]. Our results demonstrate that LOC550643 expression is not only involved in the motility and cell cycle progression of CRC cells but is also correlated with survival in patients with CRC. High LOC550643 levels correlate with an advanced pathological stage, and LOC550643 knockdown causes apoptosis in CRC cells.

Both miRNAs and lncRNAs play integral roles in regulatory gene expression and are involved in pathophysiological processes in CRC [6,36]. Studies have revealed that lncRNA modulates the growth and metastasis of colon cancer cells by sponging miRNAs or by influencing the miRNA maturation process [7,36,37]. Yu et al. reported on a novel mechanism of the interaction of CCAT2 with pre-miR-145 in the cell nucleus, which results in the suppression of the pre-miR-145 maturation process [37]. Most relevant studies have indicated that the lncRNA–miRNA interaction occurs in the cytoplasm, leading to the segregation of miRNA and the inhibition of miRNA–RNA interaction [38,39]. LOC550643 is predominantly expressed in the cytoplasm, and therefore an interaction between LOC550643 and miR-29b-2-5p may be crucial in the regulation of growth and metastasis in colon cancer cells. The miR-29 family is related to numerous diseases and implicated in tumorigenesis [40]. Notably, miR-29 can induce the epithelial–mesenchymal transition in breast and colon cancer, which may play essential roles in different stages of cancer [40]. Inoue et al. demonstrated that miR-29b expression was significantly reduced in CRC cells compared with in normal mucosa. They suggested that low miR-29b expression acted as a poor prognostic biomarker for pathological T stage, metastasis, and overall survival in patients with CRC [41]. Recent studies have indicated that miR-29b expression inhibits cell viability and stimulates cell apoptosis in human cancers, including colon cancer, through the suppression of FOLR1 expression [42,43]. The biological role of miR-29b might involve tumor suppression during colon cancer progression.

Our previous study indicated that LINC00659 knockdown and chemotherapy drugs have a synergistic effect, resulting in drug sensitivity to accelerated apoptosis in cancer cells through the modulation of PI3K/AKT signaling [44]. Liu et al. demonstrated that the differing expressions of miR-29b influence therapeutic effects in established oxaliplatin-resistant CRC cells [45]. Herein, our data indicated that LOC550643 can directly interact with miR-29b-2-5p in colon cancer cells. Furthermore, we demonstrated that LOC550643 knockdown could suppress colon cancer growth, induce apoptosis, and modulate PI3K/AKT signaling activation. However, our current results indicated that miR-29b-2-5p inhibits colon cancer growth and induces apoptosis probably not by regulating the PI3K signaling pathway. A previous study reported that miR-29b-2-5p could suppress pancreatic ductal adenocarcinoma cell growth and induce apoptosis by targeting Cbl-b to influence p53 signaling [43]. In addition, miR-29b-2-5p could suppress MEKK2 and NOTCH2 expression in lung cells by directly targeting their 3’UTR region [46]. LOC550643 shared miR-29b-2-5p binding elements with MEKK2, NOTCH2, and Cbl-b, implying that miR-29b-2-5p participated in LOC550643 regulated colon cancer growth and apoptosis through MEKK2, NOTCH2, and p53 signaling.

In addition, our previous study revealed the significant involvement of LOC550643 in breast cancer metastasis [24]. Herein, we also demonstrated that LOC550643 involved in colon cancer motility through the inhibition of filopodia formation. Furthermore, miR-29b-2-5p strongly suppressed filopodia formation through the inhibition of UNC5C expression [47]. One study reported that the overexpression of miR-29b-2-5p inhibited the EMT in the lungs and attenuated chemotherapy-induced pulmonary fibrosis in vivo [46]. Shen et al. observed that lnc556-miR-29b-2-5p and lnc865-miR-29b-2-5p inhibited pulmonary fibrosis by modulating STAT3 expression [48]. However, ectopic expression of miR-29b-2-5p did not significantly alter the migration and invasion abilities in HCT116, suggesting that miR-29b-2-5p did not involve in LOC550643 knockdown-induced suppression of colon cancer cell motility.

In summary, LOC550643 was highly expressed in CRC cells and was significantly correlated with overall survival. Our findings indicate that the LOC550643/PI3K signaling and LOC550643/miR-29b-2-5p axis might be potential therapeutic targets for CRC.

## Figures and Tables

**Table 1:** Correlation of LOC550643 expression with clinicopathological characteristics of patients with colon cancer.

Variables	LOC550643 (*n* = 446)			
	No. (%)	Mean ± SD	Median	*p*-Value
Pathology stage				
I	71 (15.9)	16.92 ± 7.27	15.97	0.029 ^a^
II	197 (44.2)	17.23 ± 7.60	16.15	
III	121 (27.1)	16.51 ± 6.81 ^c^	16.19	
IV	57 (12.8)	19.95 ± 7.59 ^c^	19.14	
pT stage				
T1	10 (2.2)	21.50 ± 9.18	19.10	0.146 ^a^
T2	71 (15.9)	16.22 ± 6.52	15.97	
T3	316 (70.9)	17.32 ± 7.38	16.53	
T4	49 (11.0)	18.15 ± 8.04	16.32	
pN stage				
N0	275 (61.7)	17.19 ± 7.49	16.15	0.778 ^a^
N1	97 (21.7)	17.80 ± 6.60	17.67	
N2	74 (16.6)	17.24 ± 8.02	16.38	
pM stage				
M0	389 (87.2)	16.95 ± 7.29	16.10	0.004 ^b^
M1	57 (12.8)	19.95 ± 7.59	19.14	

^a^*p*-value were estimated by one-way ANOVA test. ^b^*p*-value were estimated by student’s T test. ^c^*p* = 0.037.

**Table 2:** Univariate and multivariate Cox regression analyses of LOC550643 gene expression with regard to overall survival in patients with colon cancer.

Characteristic	No. (%)	OS			
		CHR (95% CI)	*p*-Value	AHR (95% CI)	*p*-Value
LOC550643	(*n* = 445)				
Low	349 (78.4)	1.00	0.001	1.00	0.006
High	96 (21.6)	2.10 (1.34–3.28)		1.90 (1.21–3.00)	

Abbreviation: OS: Overall survival; CHR, crude hazard ratio; AHR, adjusted hazard ratio. AHR were adjusted for AJCC pathological stage (II, III and IV VS. I).

**Table 3:** Gene ontology analysis of LOC550643-coexpression genes in colon cancer from TCGA database.

Gene Ontology	Function	*p*-Value
Molecular Function		
GO:0005515	protein binding	0.000160535
GO:0000146	microfilament motor activity	0.005916827
GO:0003774	cytoskeletal motor activity	0.008611768
GO:0000166	nucleotide binding	0.01087002
GO:1901265	nucleoside phosphate binding	0.010988224
GO:0036094	small molecule binding	0.014742833
GO:0043168	anion binding	0.0247294
Biological Process		
GO:0051650	establishment of vesicle localization	0.00607308
GO:0006996	organelle organization	0.00996091
GO:0051648	vesicle localization	0.013922828
GO:0030029	actin filament-based process	0.044425752
GO:0099515		0.049163434
Cellular Component	actin filament-based transport	
GO:0005829	cytosol	2.84495 × 10^−6^
GO:0005737	cytoplasm	1.19769 × 10^−5^
GO:0015629	actin cytoskeleton	0.010115212
GO:0031090	organelle membrane	0.038066502
GO:0016459	myosin complex	0.042296337
GO:0098588	bounding membrane of organelle	0.043776643

## Supplementary Materials

The following supporting information can be downloaded at: https://www.mdpi.com/article/10.3390/cells11071065/s1, Figure S1: LOC550643 expression was assessed in human cancers through the analysis of the TCGA database and GENT; Figure S2: Identification of LOC550643-coexpressed genes in colon cancer through the TCGA database and pathway enrichment analysis; Figure S3: Biological function of LOC550643 in DLD-1 cells; Figure S4: LOC550643 knockdown suppressed colon cancer cell migration and invasion abilities; Figure S5: LOC550643 localization in colon cancer cells; Figure S6: Ectopic expression of miR-29b-2-5p did not influence colon cancer cell migration and invasion abilities; Figure S7: Ectopic expression of miR-29b-2-5p impaired cell cycle and induced colon cancer apoptosis. Table S1: The antibodies used in this study

## References

[1] Bray F., Ferlay J., Soerjomataram I., Siegel R.L., Torre L.A., Jemal A. (2018). Global cancer statistics 2018: GLOBOCAN estimates of incidence and mortality worldwide for 36 cancers in 185 countries. CA Cancer J. Clin..

[2] Marley A.R., Nan H. (2016). Epidemiology of colorectal cancer. Int. J. Mol. Epidemiol. Genet..

[3] Dekker E., Tanis P.J., Vleugels J.L.A., Kasi P.M., Wallace M.B. (2019). Colorectal cancer. Lancet.

[4] Chen X., Yan C.C., Luo C., Ji W., Zhang Y., Dai Q. (2015). Constructing lncRNA functional similarity network based on lncRNA-disease associations and disease semantic similarity. Sci. Rep..

[5] Wu H.H., Lin W.C., Tsai K.W. (2014). Advances in molecular biomarkers for gastric cancer: miRNAs as emerging novel cancer markers. Expert Rev. Mol. Med..

[6] Wang J.H., Lu T.J., Kung M.L., Yang Y.F., Yeh C.Y., Tu Y.T., Chen W.S., Tsai K.W. (2020). The Long Noncoding RNA LOC441461 (STX17-AS1) Modulates Colorectal Cancer Cell Growth and Motility. Cancers.

[7] Hong W., Ying H., Lin F., Ding R., Wang W., Zhang M. (2020). lncRNA LINC00460 Silencing Represses EMT in Colon Cancer through Downregulation of ANXA2 via Upregulating miR-433-3p. Mol. Ther. Nucleic Acids.

[8] He X., Tan X., Wang X., Jin H., Liu L., Ma L., Yu H., Fan Z. (2014). C-Myc-activated long noncoding RNA CCAT1 promotes colon cancer cell proliferation and invasion. Tumour Biol..

[9] Spizzo R., Almeida M.I., Colombatti A., Calin G.A. (2012). Long non-coding RNAs and cancer: A new frontier of translational research?. Oncogene.

[10] Tseng H.W., Li S.C., Tsai K.W. (2019). Metformin Treatment Suppresses Melanoma Cell Growth and Motility Through Modulation of microRNA Expression. Cancers.

[11] Ma H., Hao Y., Dong X., Gong Q., Chen J., Zhang J., Tian W. (2012). Molecular mechanisms and function prediction of long noncoding RNA. Sci. World J..

[12] Kogo R., Shimamura T., Mimori K., Kawahara K., Imoto S., Sudo T., Tanaka F., Shibata K., Suzuki A., Komune S. (2011). Long noncoding RNA HOTAIR regulates polycomb-dependent chromatin modification and is associated with poor prognosis in colorectal cancers. Cancer Res..

[13] Wu Z.H., Wang X.L., Tang H.M., Jiang T., Chen J., Lu S., Qiu G.Q., Peng Z.H., Yan D.W. (2014). Long non-coding RNA HOTAIR is a powerful predictor of metastasis and poor prognosis and is associated with epithelial-mesenchymal transition in colon cancer. Oncol. Rep..

[14] Ge X., Chen Y., Liao X., Liu D., Li F., Ruan H., Jia W. (2013). Overexpression of long noncoding RNA PCAT-1 is a novel biomarker of poor prognosis in patients with colorectal cancer. Med. Oncol..

[15] Qi P., Xu M.D., Ni S.J., Huang D., Wei P., Tan C., Zhou X.Y., Du X. (2013). Low expression of LOC285194 is associated with poor prognosis in colorectal cancer. J. Transl. Med..

[16] Shi D., Zheng H., Zhuo C., Peng J., Li D., Xu Y., Li X., Cai G., Cai S. (2014). Low expression of novel lncRNA RP11-462C24.1 suggests a biomarker of poor prognosis in colorectal cancer. Med. Oncol..

[17] Zhai H., Fesler A., Schee K., Fodstad O., Flatmark K., Ju J. (2013). Clinical significance of long intergenic noncoding RNA-p21 in colorectal cancer. Clin. Colorectal Cancer.

[18] Yan B., Gu W., Yang Z., Gu Z., Yue X., Gu Q., Liu L. (2014). Downregulation of a long noncoding RNA-ncRuPAR contributes to tumor inhibition in colorectal cancer. Tumour Biol..

[19] Takahashi Y., Sawada G., Kurashige J., Uchi R., Matsumura T., Ueo H., Takano Y., Eguchi H., Sudo T., Sugimachi K. (2013). Amplification of PVT-1 is involved in poor prognosis via apoptosis inhibition in colorectal cancers. Br. J. Cancer.

[20] Guo Q., Zhao Y., Chen J., Hu J., Wang S., Zhang D., Sun Y. (2014). BRAF-activated long non-coding RNA contributes to colorectal cancer migration by inducing epithelial-mesenchymal transition. Oncol. Lett..

[21] Deng Q., He B., Gao T., Pan Y., Sun H., Xu Y., Li R., Ying H., Wang F., Liu X. (2014). Up-regulation of 91H promotes tumor metastasis and predicts poor prognosis for patients with colorectal cancer. PLoS ONE.

[22] Ji P., Diederichs S., Wang W., Boing S., Metzger R., Schneider P.M., Tidow N., Brandt B., Buerger H., Bulk E. (2003). MALAT-1, a novel noncoding RNA, and thymosin beta4 predict metastasis and survival in early-stage non-small cell lung cancer. Oncogene.

[23] Yin D., He X., Zhang E., Kong R., De W., Zhang Z. (2014). Long noncoding RNA GAS5 affects cell proliferation and predicts a poor prognosis in patients with colorectal cancer. Med. Oncol..

[24] Tsai K.W., Chong K.H., Li C.H., Tu Y.T., Chen Y.R., Lee M.C., Chan S.H., Wang L.H., Chang Y.J. (2021). LOC550643, a Long Non-coding RNA, Acts as Novel Oncogene in Regulating Breast Cancer Growth and Metastasis. Front. Cell Dev. Biol..

[25] Park S.J., Yoon B.H., Kim S.K., Kim S.Y. (2019). GENT2: An updated gene expression database for normal and tumor tissues. BMC Med. Genom..

[26] Raudvere U., Kolberg L., Kuzmin I., Arak T., Adler P., Peterson H., Vilo J. (2019). g:Profiler: A web server for functional enrichment analysis and conversions of gene lists (2019 update). Nucleic Acids Res..

[27] Reimand J., Kull M., Peterson H., Hansen J., Vilo J. (2007). g:Profiler—A web-based toolset for functional profiling of gene lists from large-scale experiments. Nucleic Acids Res..

[28] Naydenov N.G., Lechuga S., Huang E.H., Ivanov A.I. (2021). Myosin Motors: Novel Regulators and Therapeutic Targets in Colorectal Cancer. Cancers.

[29] Wirtz D., Konstantopoulos K., Searson P.C. (2011). The physics of cancer: The role of physical interactions and mechanical forces in metastasis. Nat. Rev. Cancer.

[30] Aghagolzadeh P., Radpour R. (2016). New trends in molecular and cellular biomarker discovery for colorectal cancer. World J. Gastroenterol..

[31] Al-Shamsi H.O., Alhazzani W., Wolff R.A. (2015). Extended RAS testing in metastatic colorectal cancer-Refining the predictive molecular biomarkers. J. Gastrointest. Oncol..

[32] He R.Z., Luo D.X., Mo Y.Y. (2019). Emerging roles of lncRNAs in the post-transcriptional regulation in cancer. Genes Dis..

[33] Luo J.Z., Qin L., Zhang L.J. (2020). Expression and function of long non-coding RNA LINC01420 in thyroid cancer. Oncol. Lett..

[34] Yang L., Tang Y., He Y., Wang Y., Lian Y., Xiong F., Shi L., Zhang S., Gong Z., Zhou Y. (2017). High Expression of LINC01420 indicates an unfavorable prognosis and modulates cell migration and invasion in nasopharyngeal carcinoma. J. Cancer.

[35] Zhai H., Zhang X., Sun X., Zhang D., Ma S. (2020). Long Non-coding RNA LINC01420 Contributes to Pancreatic Cancer Progression Through Targeting KRAS Proto-oncogene. Dig. Dis. Sci..

[36] Chen W.S., Chen T.W., Yang T.H., Hu L.Y., Pan H.W., Leung C.M., Li S.C., Ho M.R., Shu C.W., Liu P.F. (2013). Co-modulated behavior and effects of differentially expressed miRNA in colorectal cancer. BMC Genom..

[37] Yu Y., Nangia-Makker P., Farhana L., Majumdar A.P.N. (2017). A novel mechanism of lncRNA and miRNA interaction: CCAT2 regulates miR-145 expression by suppressing its maturation process in colon cancer cells. Mol. Cancer.

[38] Chen S., Shen X. (2020). Long noncoding RNAs: Functions and mechanisms in colon cancer. Mol. Cancer.

[39] Goodall G.J., Wickramasinghe V.O. (2021). RNA in cancer. Nat. Rev. Cancer.

[40] Alizadeh M., Safarzadeh A., Beyranvand F., Ahmadpour F., Hajiasgharzadeh K., Baghbanzadeh A., Baradaran B. (2019). The potential role of miR-29 in health and cancer diagnosis, prognosis, and therapy. J. Cell Physiol..

[41] Inoue A., Yamamoto H., Uemura M., Nishimura J., Hata T., Takemasa I., Ikenaga M., Ikeda M., Murata K., Mizushima T. (2015). MicroRNA-29b is a Novel Prognostic Marker in Colorectal Cancer. Ann. Surg. Oncol..

[42] Fu Q., Zhang J., Huang G., Zhang Y., Zhao M., Zhang Y., Xie J. (2020). microRNA-29b inhibits cell growth and promotes sensitivity to oxaliplatin in colon cancer by targeting FOLR1. Biofactors.

[43] Li C., Dong Q., Che X., Xu L., Li Z., Fan Y., Hou K., Wang S., Qu J., Xu L. (2018). MicroRNA-29b-2-5p inhibits cell proliferation by directly targeting Cbl-b in pancreatic ductal adenocarcinoma. BMC Cancer.

[44] Tsai K.W., Lo Y.H., Liu H., Yeh C.Y., Chen Y.Z., Hsu C.W., Chen W.S., Wang J.H. (2018). Linc00659, a long noncoding RNA, acts as novel oncogene in regulating cancer cell growth in colorectal cancer. Mol. Cancer.

[45] Liu H., Cheng X.H. (2018). MiR-29b reverses oxaliplatin-resistance in colorectal cancer by targeting SIRT1. Oncotarget.

[46] Xu Q., Cheng D., Liu Y., Pan H., Li G., Li P., Li Y., Sun W., Ma D., Ni C. (2021). LncRNA-ATB regulates epithelial-mesenchymal transition progression in pulmonary fibrosis via sponging miR-29b-2-5p and miR-34c-3p. J. Cell Mol. Med..

[47] Sunkavalli U., Aguilar C., Silva R.J., Sharan M., Cruz A.R., Tawk C., Maudet C., Mano M., Eulalio A. (2017). Analysis of host microRNA function uncovers a role for miR-29b-2-5p in Shigella capture by filopodia. PLoS Pathog..

[48] Shen K., Li R., Zhang X., Qu G., Li R., Wang Y., Liu B., Lv C., Li M., Song X. (2021). Acetyl oxygen benzoate engeletin ester promotes KLF4 degradation leading to the attenuation of pulmonary fibrosis via inhibiting TGFbeta1-smad/p38MAPK-lnc865/lnc556-miR-29b-2-5p-STAT3 signal pathway. Aging.

