# Supplementary material for: Long Noncoding RNA LOC550643 Acts as an Oncogene in the Growth Regulation of Colorectal Cancer Cells
Source: Cells. 2022 Mar 22;11(7):1065. doi: 10.3390/cells11071065 (PMC8997465; doi:10.3390/cells11071065)
Supplement: Supplementary file 1 [file cells-11-01065-s001.zip › cells-1610610-supplementary.pdf]

Supplementary Figures and Tables

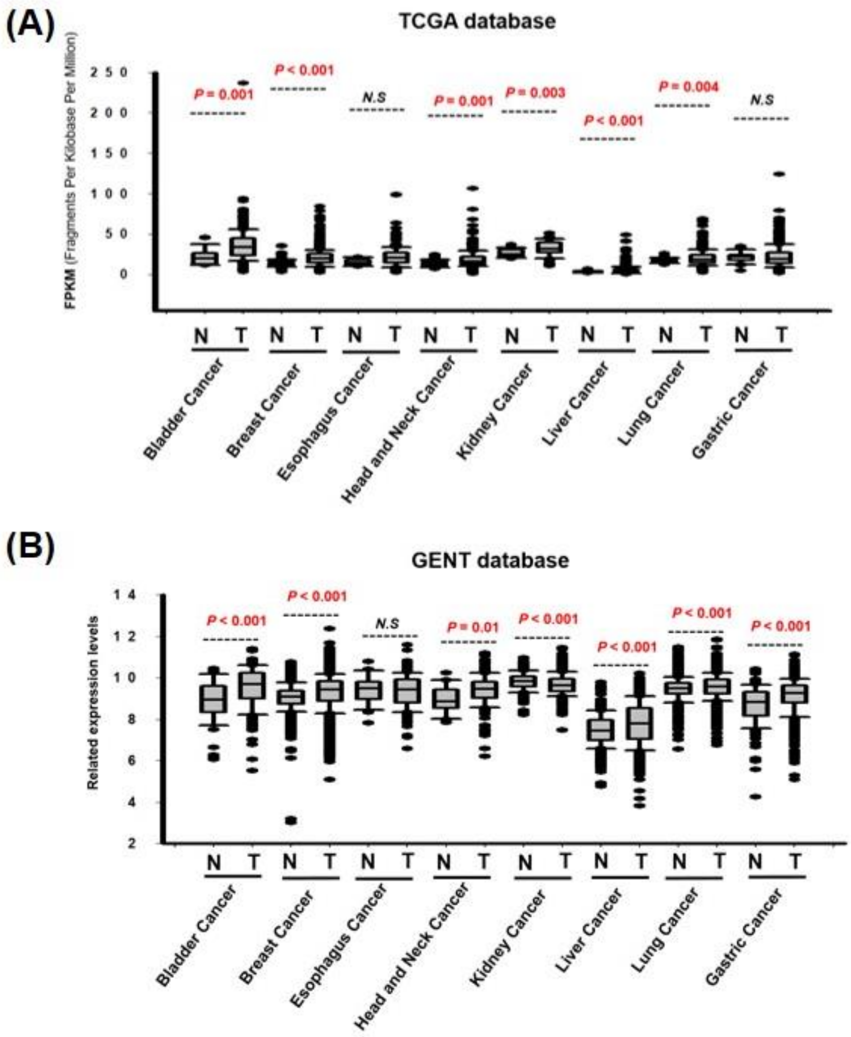

Supplementary Figure S1. LOC550643 expression was assessed in human cancers through the analysis of the TCGA database and GENT.

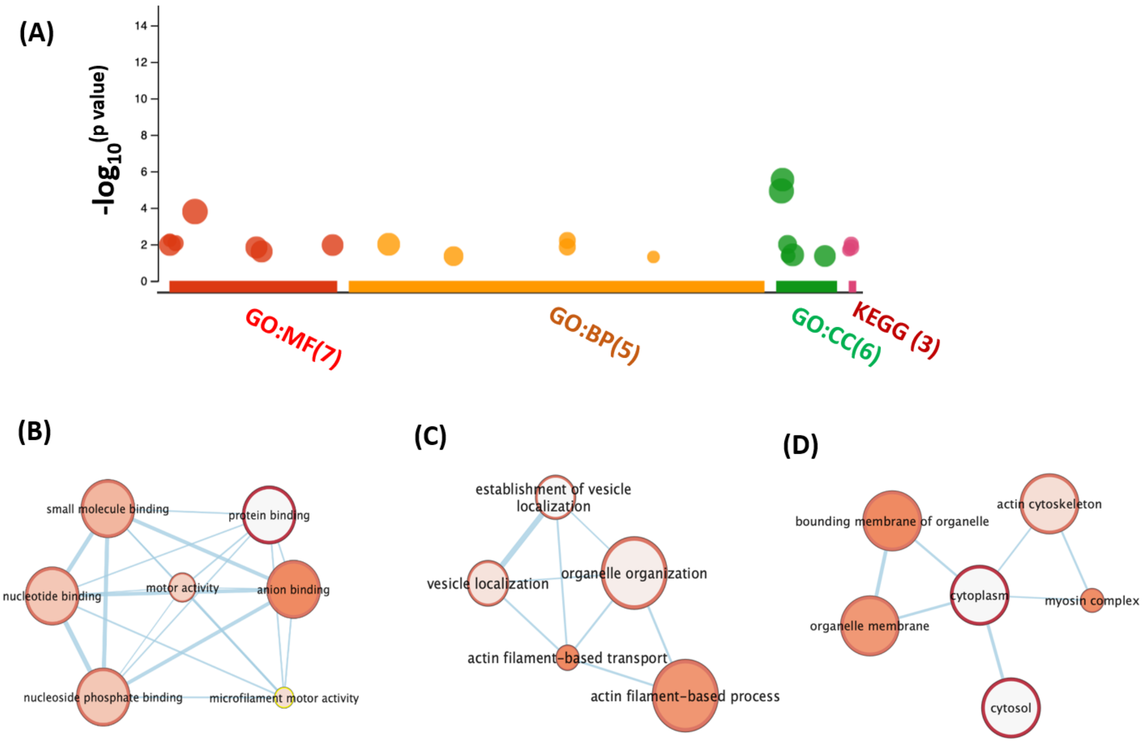

Supplementary Figure S2 Identification of LOC550643-coexpressed genes in colon cancer through the TCGA database and pathway enrichment analysis. (A) the LOC550643 co-expressed genes were analyzed by g: Profiler approach. The relative signaling pathways were signaling enriched in (B) Molecular Function, (C) Biological Process and (C) Cellular Component.

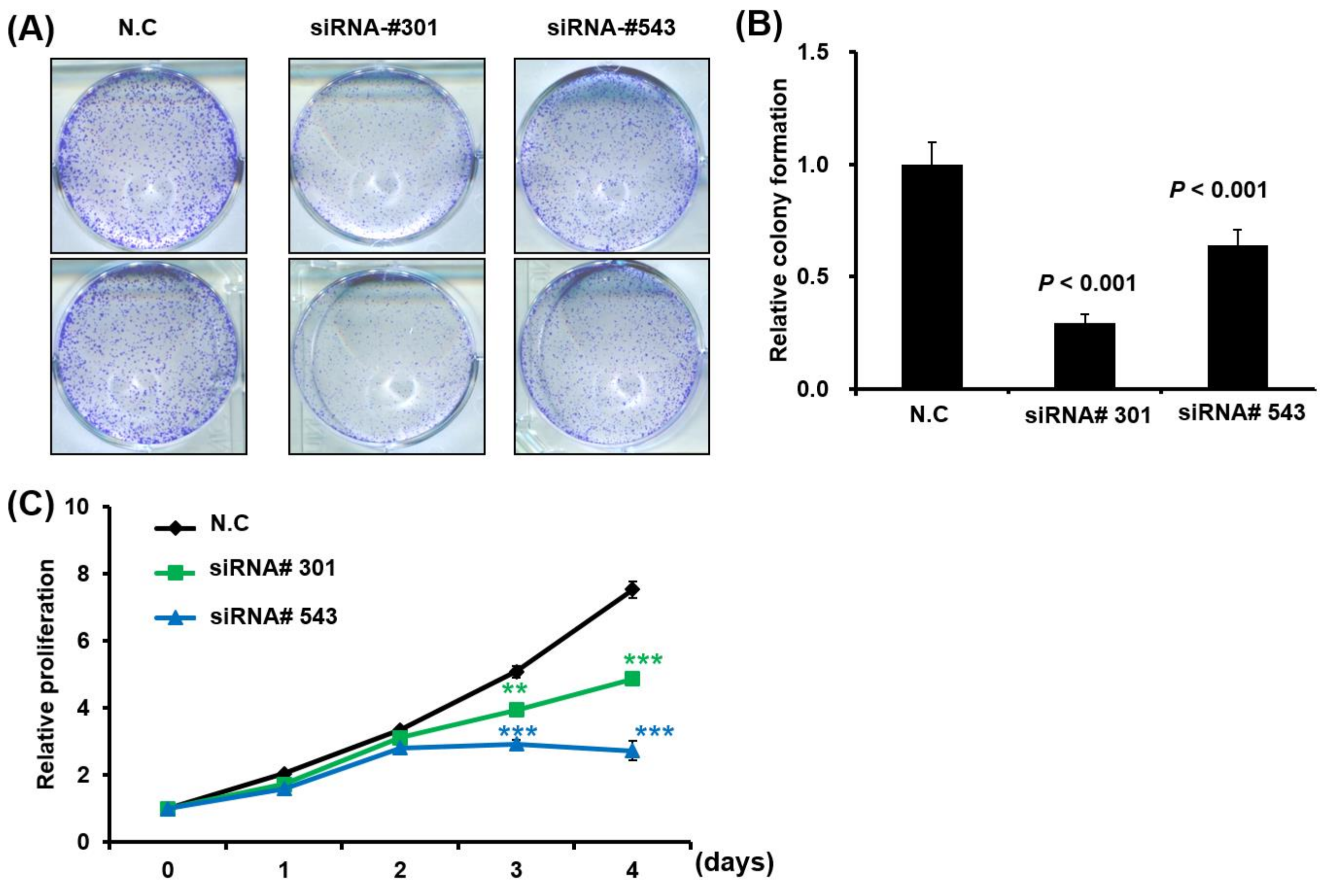

Supplementary Figure S3. Biological function of LOC550643 in DLD-1 cells. (A) Colony formation ability of DLD-1 cells with LOC550643 knockdown after their transfection with siRNA#301, siRNA#543, or a scrambled control. (B) Relative colony formation ability was quantified. (C) A cell proliferation assay after their transfection with siRNA#301, siRNA#543, or a scrambled control. (*$p < 0.05$, **$p < 0.01$, ***$p < 0.001$).

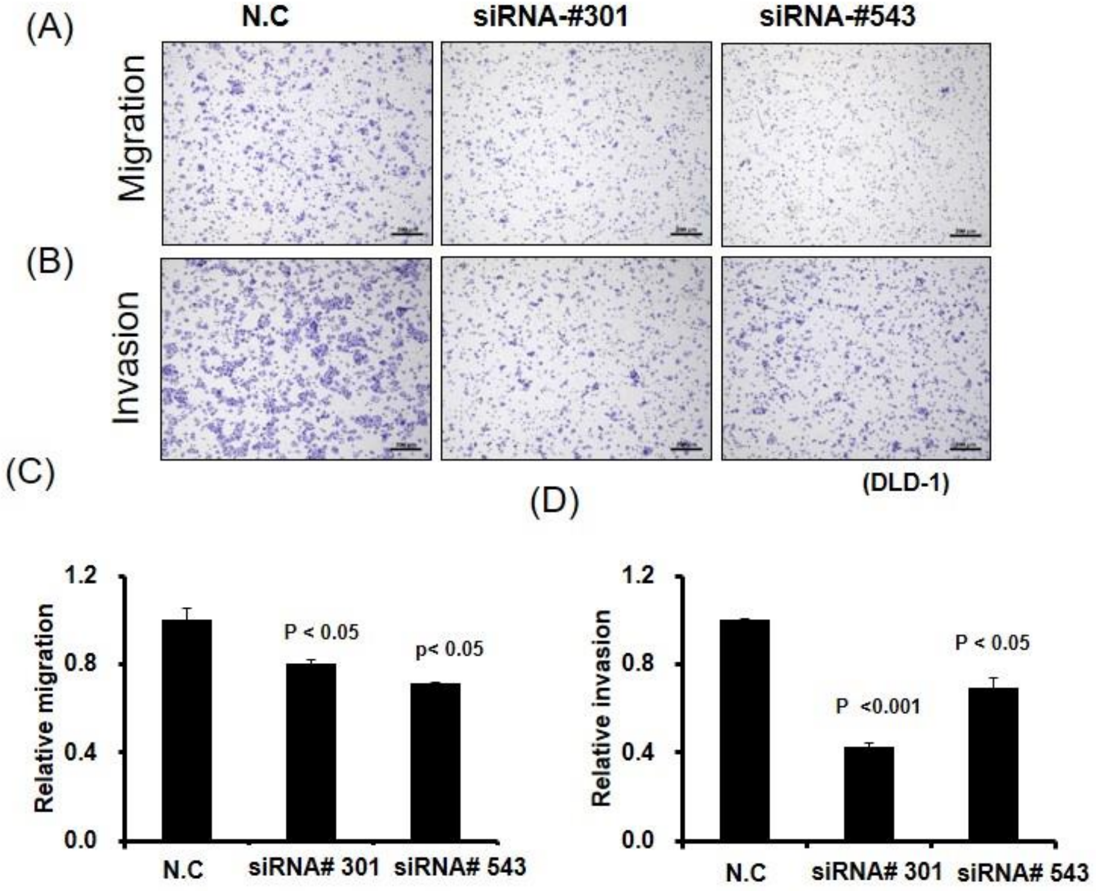

Supplementary Figure S4. LOC550643 knockdown suppressed colon cancer cell migration and invasion abilities. (A, B) The migration/invasion ability of DLD-1 cells with LOC550543 after siRNA transfection was assessed through the transwell migration assay. (C, D) Relative migration/invasion ability was quantified using Ascent software.

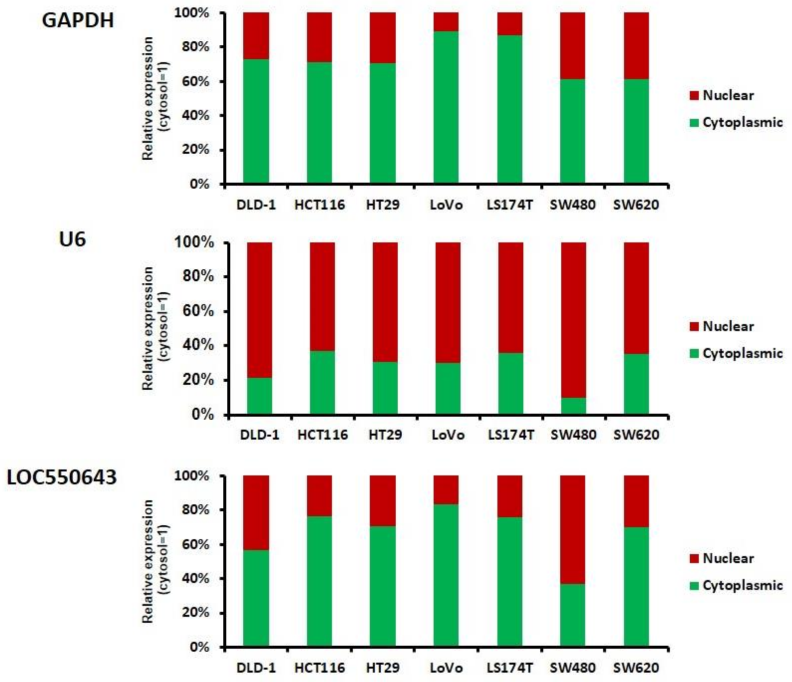

Supplementary Figure S5. LOC550643 localization in colon cancer cells. After nuclear and cytosolic separation, total RNA was extracted. Cytosolic and nuclear total RNA was subjected to cDNA conversion, and then LOC550643 expression in the cytoplasm and nuclei of LoVo, HT29, HCT116, LS174T, Lovo, SW480, SW620 and DLD-1 cells was assayed through real-time PCR. Furthermore, the expression levels of GAPDH, U6, and LOC550643 were examined in the nucleus and cytoplasm of the same cell lines through real-time PCR. GAPDH and U6 were used as cytosolic and nuclear markers, respectively.

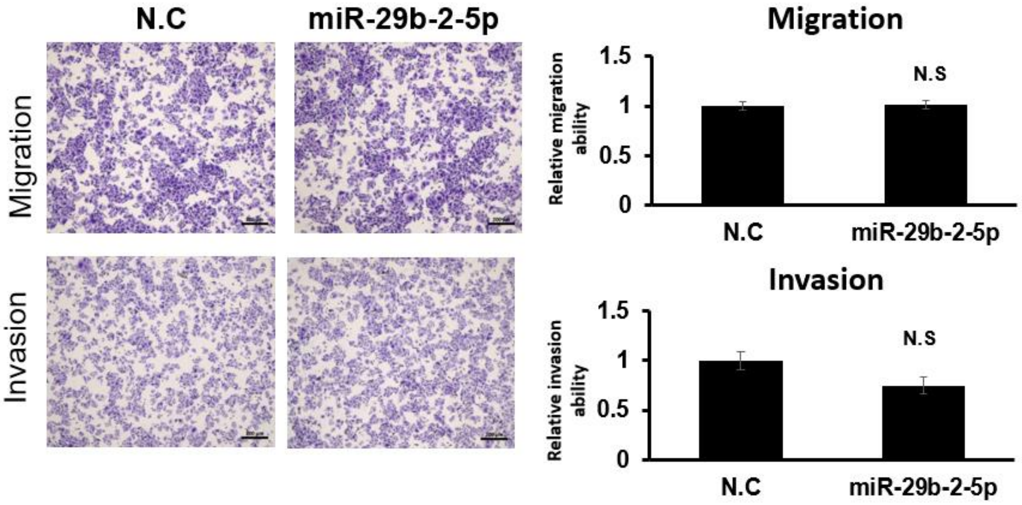

Supplementary Figure S6. Ectopic expression of miR-29b-2-5p did not influence colon cancer cell migration and invasion abilities. (A, B) miR-29b-2-5p mimics was transfected into HCT116 cells, and the migration and invasion abilities were assessed through the transwell migration/invasion assay. (C, D) Relative migration and invasion abilities were further quantified.

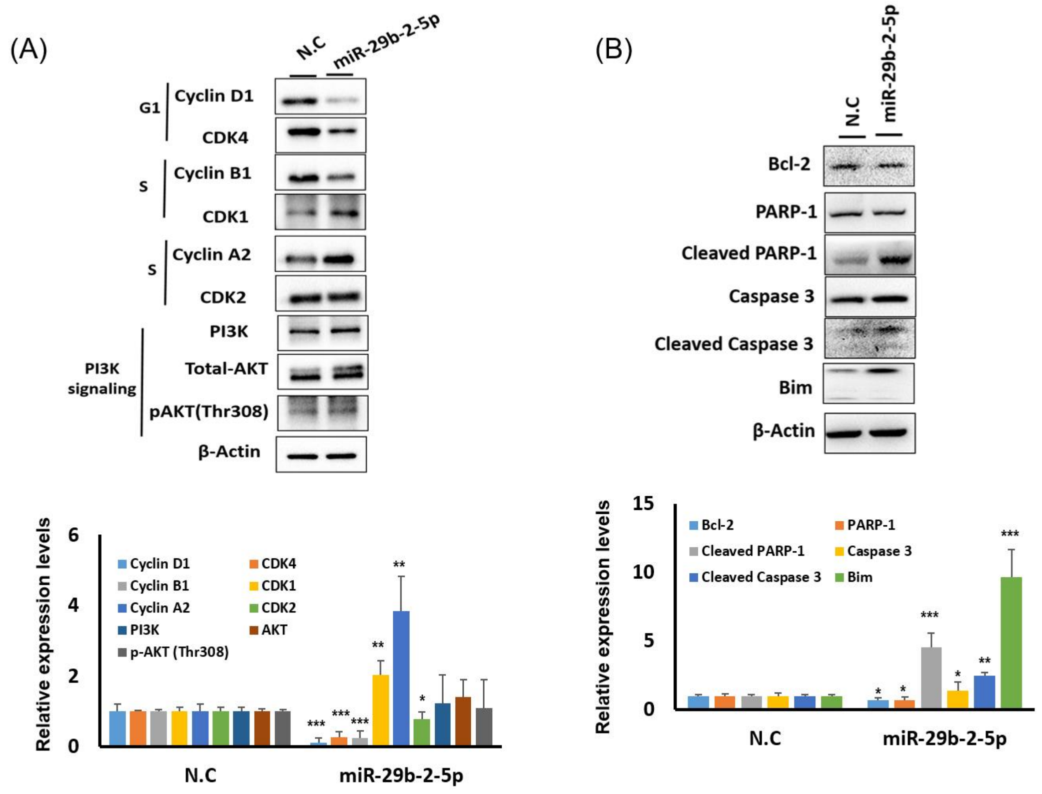

Supplementary Figure S7. Ectopic expression of miR-29b-2-5p impaired cell cycle and induced colon cancer apoptosis. (A) Western blotting was conducted to assess the expression levels of cell cycle–related and PI3K signaling-related proteins in HCT116 cells with miR-29b-2-5p overexpression. Relative expression was further quantification (below panel). (B) Apoptosis-associated protein was assayed in HCT116 cells with miR-29b-2-5p overexpression. Relative expression was further quantification (below panel). (*$p < 0.05$, **$p < 0.01$, ***$p < 0.001$).

Supplementary Table S1. The antibodies used in this study

Antibody name	1st Ab Dilution	MW (kDa)	Company	Host	2nd Ab Dilution
CDK1	1:200	34, 27	10762-1-AP, proteintech	Rabbit	1:5000
CDK4	1:1000	34	MA5-12984, ThermoFisher	Mouse	1:5000
CDK2	1:1000	34	MA5-17052, ThermoFisher	Mouse	1:5000
CyclinA2	1:500	56	18202-1-AP, proteintech	Rabbit	1:5000
CyclinB1	1:500	55-60	55004-1-AP, proteintech	Rabbit	1:5000
CyclinD1	1:200	36	MA5-16356, ThermoFisher	Rabbit	1:5000
P21	1:1000	21	#2947, Cell signaling Technology	Rabbit	1:5000
P27	1:500	27	25614-1-AP, proteintech	Rabbit	1:5000
PI3K	1:1000	85	#4292, Cell signaling Technology	Rabbit	1:5000
Total-AKT	1:1000	60	#4691, Cell signaling Technology	Rabbit	1:5000
pAKT(Ser473)	1:1000	60	#4060, Cell signaling Technology	Rabbit	1:5000
pAKT(Thr308)	1:1000	60	#4056, Cell signaling Technology	Rabbit	1:5000
PARP-1	1:200	116, 89	C-8007, Santa Cruz Biotechnology	Mouse	1:5000
Caspase 3	1:1000	17, 19, 35	#9662, Cell signaling Technology	Rabbit	1:5000
Bim	1:1000	12, 15, 23	#2933, Cell signaling Technology	Rabbit	1:5000
Bcl-2	1:1000	26	#4223, Cell signaling Technology	Rabbit	1:5000
β-actin (ACTB)	1:5000	43	MAB1501, Millipore	Mouse	1:5000

*MW: Molecular Weight
